# Factors associated with psychological distress, fear and coping strategies during the COVID-19 pandemic in Australia

**DOI:** 10.1186/s12992-020-00624-w

**Published:** 2020-10-08

**Authors:** Muhammad Aziz Rahman, Nazmul Hoque, Sheikh M. Alif, Masudus Salehin, Sheikh Mohammed Shariful Islam, Biswajit Banik, Ahmed Sharif, Nashrin Binte Nazim, Farhana Sultana, Wendy Cross

**Affiliations:** 1 School of Health, Federation University Australia, 100 Clyde Road, Berwick, Victoria 3806 Australia; 2 Australian Institute of Primary Care and Ageing, La Trobe University, Plenty Road and Kingsbury Drive, Melbourne, Victoria 3086 Australia; 3 Bangladesh Medical Society of Victoria (BMSV), Melbourne, VIC 3000 Australia; 4 Emerald Medical Centre, 1 Murphys Way, Emerald, Victoria 3782 Australia; 5 School of Public Health and Preventive Medicine, Monash University, 553 St Kilda Road, Melbourne, Victoria 3004 Australia; 6 Institute for Physical Activity and Nutrition, Deakin University, 221 Burwood Highway, Burwood, Victoria 3125 Australia; 7 Station Street Clinic, 34 Station Street, Pakenham, Burwood, Victoria 3810 Australia; 8 Bangladesh Institute of Family Medicine and Research, University of Science & Technology Chittagong, Zakir Hossain Road, Foy’s Lake, Khulshi, Chittagong, 4202 Bangladesh; 9 Greenvale Medical Centre, 1/11 Greenvale Drive, Greenvale, Burwood, Victoria 3059 Australia; 10 Telstra Health, 222 Lonsdale Street, Melbourne, Victoria 3000 Australia

**Keywords:** COVID-19, Coronavirus, Mental health, Psychological distress, Coping, Resilience

## Abstract

**Background:**

The COVID-19 pandemic disrupted the personal, professional and social life of Australians with some people more impacted than others.

**Objectives:**

This study aimed to identify factors associated with psychological distress, fear and coping strategies during the COVID-19 pandemic in Australia.

**Methods:**

A cross-sectional online survey was conducted among residents in Australia, including patients, frontline health and other essential service workers, and community members during June 2020. Psychological distress was assessed using the Kessler Psychological Distress Scale (K10); level of fear was assessed using the Fear of COVID-19 Scale (FCV-19S); and coping strategies were assessed using the Brief Resilient Coping Scale (BRCS). Logistic regression was used to identify factors associated with the extent of psychological distress, level of fear and coping strategies while adjusting for potential confounders.

**Results:**

Among 587 participants, the majority (391, 73.2%) were 30–59 years old and female (363, 61.8%). More than half (349, 59.5%) were born outside Australia and two-third (418, 71.5%) completed at least a Bachelor’s degree. The majority (401, 71.5%) had a source of income, 243 (42.3%) self-identified as a frontline worker, and 335 (58.9%) reported financial impact due to COVID-19. Comorbidities such as pre-existing mental health conditions (AOR 3.13, 95% CIs 1.12–8.75), increased smoking (8.66, 1.08–69.1) and alcohol drinking (2.39, 1.05–5.47) over the last four weeks, high levels of fear (2.93, 1.83–4.67) and being female (1.74, 1.15–2.65) were associated with higher levels of psychological distress. Perceived distress due to change of employment status (4.14, 1.39–12.4), alcohol drinking (3.64, 1.54–8.58), providing care to known or suspected cases (3.64, 1.54–8.58), being female (1.56, 1.00–2.45), being 30–59 years old (2.29, 1.21–4.35) and having medium to high levels of psychological distress (2.90, 1.82–5.62) were associated with a higher level of fear; while healthcare service use in the last four weeks was associated with medium to high resilience.

**Conclusions:**

This study identified individuals who were at higher risk of distress and fear during the COVID-19 pandemic specifically in the State of Victoria, Australia. Specific interventions to support the mental wellbeing of these individuals should be considered in addition to the existing resources within primary healthcare settings.

## Introduction

The coronavirus disease (COVID-19) had affected more than 213 countries and territories around the world with more than 28 million cases and nearly one million deaths as of mid-Sep-2020 [1]. In the same time frame, Australia reported over 25,000 confirmed cases and about 800 deaths from COVID-19 [2]. Although the case fatality rate was low in Australia compared to other developed countries such as USA or UK, people were distressed due to the nature of transmission (i.e. through direct or indirect contact) and the rapid spread within the communities, which radically changed regular lifestyles for most Australians [3].

In order to limit the spread of COVID-19, the Australian Government introduced physical distancing rules including restrictions on social gatherings, strict lockdowns, and border closures with a pause on all social, cultural and sporting activities [2]. These restrictions were likely to have tremendous impact on the social, psychological and economic wellbeing of Australians living through this pandemic [4]. Strict border closures and physical distancing measures played a crucial role in reducing the spread of community transmission resulting in a ‘flattening of the curve’ from late March to mid-June 2020. However, Australia has faced a second wave of pandemic with the highest increase of daily case number of more than 700 in the state of Victoria in early August 2020 [2]. Many small businesses were closed due to the imposed restrictions from late March 2020 and the unemployment rate increased to 15% in June 2020 compared to 9% in June 2019 [4, 5]. Stricter restrictions and night-time curfews were implemented in the State of Victoria solely to curb the spread of infection. The continued impact of restrictions and the uncertainty around going back to normal were likely to affect personal and social life as well as mental wellbeing of all Australians [4]. Ongoing restrictions were likely to impact physical health as well, specifically those with chronic diseases. Health care seeking behaviour was also affected by the COVID-19 pandemic, as patients limited their in-person visits to their general practitioners (GPs) to avoid the possible risk of transmission from suspected/asymptomatic cases of COVID-19 and have started to access telehealth facilities [6]. Moreover, social isolation, along with uncertainty in employment status could have triggered risky behaviours such as increased smoking and alcohol intake [4].

Frontline workers including the health care workers were at increased risk of infection during the COVID-19 pandemic. Evidence suggests that frontline healthcare workers, who were directly involved in the collection of samples, diagnosis, treatment, and care of patients during an outbreak, were also at higher risk of developing psychological distress and mental health symptoms [7]. Anxiety, distress, depression, fear of spread of infection to family, friends and colleagues, anger and confusion were some of the immediate psychological impacts documented among frontline healthcare workers [4, 8]. Ongoing restrictions, uncertainty of returning to normal life and deaths that were avoidable under usual circumstances were also likely to increase people’s risk of developing long-term mental health issues [9]. It was, therefore, important to understand the extent of the mental health burden in communities during the COVID-19 pandemic in Australia. This study will assist in designing appropriate psychosocial interventions and will provide a baseline against which such interventions could be assessed.

Evidence examining factors associated with psychological distress and fear due to the COVID-19 pandemic in Australia was limited. A recently published study suggested that increased psychological distress was more common among middle-aged single women and mothers, and those in lower-income categories [4]. Prior evidence suggested that lack of sleep, increased smoking and alcohol intake were associated with higher levels of depression, anxiety and stress during the pandemic [8]. Another study found that psychological distress was associated with self-reported mood disorder and lifestyle changes [3]. However, evidence-based evaluations on psychological distress, fear and coping strategies were relatively scarce [8]. To address this gap, the aim of the current study was to (a) Assess the extent of psychological distress and the level of fear of COVID-19 among residents in Australia, and (b) Identify coping strategies and key factors associated with psychological distress, fear and coping during the pandemic period.

## Materials and methods

### Study design and settings

This anonymous cross-sectional study was hosted on the online platform Qualtrics and distributed using social media (Facebook, Messenger, Twitter and LinkedIn), text messages and emails. Participants were recruited from GP and Allied Healthcare settings, and community groups via the online platform across all states and territories in Australia between 1st and 30th June 2020.

### Study population

Australian residents aged ≥18 years and capable of responding to an online questionnaire in English were invited to participate. Study participants included (a) patients who attended a general practice or an allied healthcare setting, either for face-to-face or telehealth consultations in the last four weeks irrespective of COVID-19 symptoms; (b) Frontline workers (full time, part time or casual) who were in contact with patients/clients (with known or unknown status of COVID-19) in the last four weeks, and (c) Community members, who did not consult with GP or allied health service providers in the last four weeks.

### Sampling

All participants fulfilling the inclusion criteria were invited to participate. Sample size was calculated using OpenEpi. Considering 25,464,116 as the population of Australia [10], the prevalence of lifetime mental health issues amongst Australians at 45% [11], at 95% confidence intervals and 80% power, the estimated minimum sample size was 381. Snowball sampling was used to recruit the study participants.

### Data collection

An online link of the web-based questionnaire was developed using Qualtrics from the Federation University Australia after the ethics approval. The initial eligibility question was related to age and place of residence, which on fulfilling, participants had the opportunity to consent and commence the main study questionnaire. The anonymous questionnaire was introduced and the invitation with the online link and QR code was shared on social media platforms, emails, and text messages. No information which could potentially identify any study participants such as name, residential address, patient identification number were collected. Patients, who visited GP clinics or Allied Healthcare settings, were informed about the study by either receptionists or healthcare professionals. Study details with the survey link and QR code were also displayed at those settings, therefore, any patient willing to participate in the study could access the link by scanning the code or accessing the survey link. However, neither health professionals nor investigators were aware of the patients who participated in the study. Patients had the freedom to complete the questionnaire at their convenience at home or while waiting to visit health professionals, and the online questionnaire did not collect any identifying information from patients.

### Study tool

A structured online survey questionnaire was used to collect data from participants, which was developed based on evidence from studies published previously [12–15]. Data were collected regarding socio-demographics, which included age, gender, state, postcode, living with or without family members, country of birth, and completed level of education. Data regarding profession and current employment condition including the impact of COVID-19 on occupation, self-identification as a frontline health or other essential service worker were also collected. In addition, the collected data included self-reported co-morbidities (cardiac diseases, stroke, hypertension, hyperlipidaemia, diabetes, cancer, chronic respiratory illness, psychological/mental health problems), behavioural risk factors (smoking and alcohol intake), health service utilization in the last four weeks including type of service providers and access to mental health resources, and history of exposure to COVID-19 including diagnosis and testing. Psychological impact was assessed by the Kessler Psychological Distress Scale (K10) [16], fear was assessed by the Fear of COVID-19 scale (FCV-19S) [17], and coping strategies were assessed by the Brief Resilient Coping Scale (BRCS) [18]. K10 was found to be a valid and reliable tool, with wide applications in research and clinical practice [16, 19, 20]; FCV-19S was developed more recently in response to the global pandemic of COVID-19, which was validated and tested for reliability in a few recent studies [17, 21, 22]; validity and reliability had also been tested for BRCS in earlier studies [18, 23, 24]. K10 has 10 items and response to each item in the questionnaire was measured using a 5-point Likert scale (none, a little, sometimes, most of the time, all the time). All items were scored, and the total score categorised into low (score 10–15), moderate (score 16–21), high (score 22–29) and very high (score 30–50). FCV-19S has seven items and the response to each item was also measured using a 5-point Likert scale (strongly disagree, somewhat disagree, neither agree nor disagree, somewhat agree, strongly agree), with scores categorised into low (score 7–21) and high (score 22–35). BRCS has four items and responses were collected again using a 5-point Likert scale (does not describe me at all, does not describe me, neutral, describes me, describes me very well), with scores similarly categorised into low (score 4–13), medium (score 14–16) and high (score 17–20) resilient copers. Following ethics approval, the questionnaire was pre-tested and finalised with feedback from the research team.

### Data analyses

The database was downloaded from Qualtrics and analysed using SPSS v.25 and STATA v.12. Descriptive analyses were used to describe the study variables. Mean and standard deviations were calculated for the continuous variable (age) and for each scale (K10, FCV-19S and BRCS). To conduct inferential analyses, K10 was defined into low (score 10–15) and moderate to very high (score 16–50), and BRCS was also defined into low (score 4–13) and medium to high (score 14–20) resilient copers. Participants, who took < 1 min to complete the questionnaire, were excluded from the analyses. Initially, the factors associated with psychological distress were identified by comparing low and moderate to very high distress on the K10 scale, factors associated with fear of COVID-19 were identified by comparing low and high fear on the FCV-19S scale, and factors associated with coping were identified by comparing low and medium to high resilient copers on the BRCS scale using Chi-square test. Statistical significance was determined by *p* < 0.05. Binary logistic regression was used to assess the strength of association, which yielded odds ratio (OR) and 95% confidence intervals (CIs). Multivariate analyses were conducted by adjusting the socio-demographic variables (age, gender, living status, country of birth, education, and employment status), and presented as adjusted OR (AOR) with 95% CI.

### Ethics

Ethics approval was obtained from the Human Research Ethics Committee (HREC) at Federation University Australia (B20–036). Data were collected anonymously and delinked for this online survey. Information on contacting BeyondBlue hotline (free of cost and 24/7 availability) was included in the participant information sheet for any respondent feeling distressed while completing the study questionnaire.

## Results

A total of 587 individuals participated in this study, the majority hailing from Victoria (88.2%). Mean age (±SD) of the participants was 41.3 (±12.5) years and 61.8% were females. Most of them (77.1%) lived with partners and/or children. More than half of the participants (59.5%) were born outside of Australia. More than two-thirds of the study population (418, 71.5%) completed at least a Bachelor course. The majority had a source of income (401/561, 71.5%) during this pandemic, and 76.3% (160/561) were perceived to have moderate to a great deal of distress due to a change of employment status due to COVID-19. Around 59% of participants also reported financial impact due to COVID-19. More than one-third (42.3%) identified themselves as frontline or essential service workers, including doctors (11%), nurses (9%) and medical receptionists (3.8%) (Table 1).

**Table 1:** Characteristics of the study population

Characteristics	Total, ***n***(%)
Total study participants	587
**Age (in years)**	**534**
Mean (±SD)	41.3 (12.5)
Range	18 to 77
**Age groups**	**534**
18–29 years	102 (19.1)
30–59 years	391 (73.2)
≥60 years	41 (7.7)
**Gender**	**587**
Male	221 (37.6)
Female	363 (61.8)
Others	3 (0.5)
**Location in Australia**	**585**
Victoria	516 (88.2)
New South Wales	22 (3.8)
Queensland	10 (1.7)
Western Australia	9 (1.5)
Australian Capital Territory	8 (1.4)
Northern Territory	8 (1.4)
South Australia	6 (1.0)
Tasmania	6 (1.0)
**Living status**	**581**
Live without family members (on your own/shared house/others)	133 (22.9)
Live with family members (partner and/or children)	448 (77.1)
**Born in Australia**	**587**
Yes	238 (40.5)
No	349 (59.5)
**Completed level of education**	**585**
Grade 1–12	76 (13.0)
Trade/Certificate/Diploma	91 (15.6)
Bachelor and above	418 (71.5)
**Current employment condition**	**561**
Unemployed/Home duties	67 (11.9)
Jobs affected by COVID-19 (lost job/working hours reduced/afraid of job loss)	93 (16.6)
Have an income source (employed/Government benefits)	401 (71.5)
**Perceived distress due to change of employment status**	**160**
Moderate to a great deal	122 (76.3)
A little to none	38 (23.8)
**Self-identification as a frontline or essential service worker**	**574**
Yes	243 (42.3)
No	331 (57.7)
**COVID-19 impacted financial situation**	**569**
Yes	335 (58.9)
No	234 (41.1)
**Co-morbidities**	**571**
No	285 (49.9)
Psychiatric/Mental health issues	41 (7.2)
Other co-morbidities*	245 (42.9)
**Smoking**	**441**
Ever smoker (Daily/Non-daily/Ex)	59 (13.4)
Never smoker	382 (86.6)
**Increased smoking over the last 4 weeks**	**59**
Yes	25 (42.4)
No	34 (57.6)
**Current alcohol drinking (last 4 weeks)**	**568**
Yes	210 (37.0)
No	358 (63.0)
**Increased alcohol drinking over the last 4 weeks**	**207**
Yes	65 (31.4)
No	142 (68.6)
**Provided care to a family member/patient with known/suspected case of COVID-19**	**566**
Yes	101 (17.8)
No	465 (82.2)
**Experience related to COVID-19 pandemic (multiple responses possible)**	**566**
I have been self-isolating prior to receiving negative result for COVID-19	73 (12.4)
I had recent overseas travel history and was in self-quarantine	9 (1.5)
No known exposure to COVID-19	470 (80.1)
**Healthcare service use in the last 4 weeks**	**563**
Visited healthcare providers in person	64 (11.4)
Telehealth consultation with healthcare providers/National helpline	50 (8.9)
No	449 (79.8)
**Healthcare service use to overcome COVID-19 related stress in the last 4 weeks**	**549**
Yes	38 (6.9)
No	511 (93.1)
**Type of healthcare service used to overcome COVID-19 related stress in the last 4 weeks**	**38**
Consulted a GP	12 (31.6)
Consulted a Psychologist	6 (15.8)
Consulted a Psychiatrist	3 (7.9)
Used specialised mental healthcare settings	1 (2.6)
Used mental health resources	2 (5.3)
Used mental health resources available through media	6 (15.8)
Used mental health support services	8 (21.1)

^*^ *Cardiac diseases/Stroke/Hypertension/Hyperlipidaemia/Diabetes/Cancer/Chronic respiratory illness*

Half of the study participants did not report any co-morbidity, and 7.2% reported having pre-existing psychiatric or mental health issues. The majority of the participants (86.6%) were never smokers and more than half (63%) did not report drinking alcohol in the last four weeks. However, of those who smoked and drink alcohol, 42.4 and 31.4% reported increased smoking and alcohol drinking in the last four weeks, respectively. One in five participants (17.8%) provided direct or indirect care to the family members or patients with a known or suspected case of COVID-19, and 13.9% reported self-isolating prior to receiving negative test results or had an overseas travel history. One in ten participants (11.4%) visited healthcare providers in person, and 8.9% had telehealth consultation in the last four weeks for any reason, while 6.9% had a consultation due to COVID-19 related stress. The latter group who consulted to overcome stress related to COVID-19 used a range of service providers including GPs (31.6%), mental health support services (21.1%), psychologists (15.8%) and psychiatrists (7.9%) (Table 1). Almost a third of the participants experienced high to very high levels of psychological distress (33%) and high levels of fear of COVID-19 (31.9%) with almost all of them (97.3%) being low resilient copers (Tables 2, 3, and 4).

**Table 2:** Level of psychological distress among the study participants

Anxiety and Depression Checklist (K10) (last 4 weeks)	Total, ***n***(%)
**About how often did you feel tired out for no good reason?**	**546**
None	156 (28.6)
A little	118 (21.6)
Sometime	184 (33.7)
Most of the time	72 (13.2)
All the time	16 (2.9)
**About how often did you feel nervous?**	**547**
None	127 (23.2)
A little	157 (28.7)
Sometime	185 (33.8)
Most of the time	70 (12.8)
All the time	8 (1.5)
**About how often did you feel so nervous that nothing could calm you down?**	**546**
None	357 (65.4)
A little	96 (16.4)
Sometime	76 (13.9)
Most of the time	16 (2.9)
All the time	1 (0.2)
**About how often did you feel hopeless?**	**547**
None	258 (47.2)
A little	151 (27.6)
Sometime	98 (17.9)
Most of the time	35 (6.4)
All the time	5 (0.9)
**About how often did you feel restless or fidgety?**	**547**
None	178 (32.5)
A little	177 (32.4)
Sometime	138 (25.2)
Most of the time	49 (9.0)
All the time	5 (0.9)
**About how often did you feel so restless you could not sit still?**	**547**
None	341 (62.3)
A little	117 (21.4)
Sometime	73 (13.3)
Most of the time	15 (2.7)
All the time	1 (0.2)
**About how often did you feel so depressed?**	**546**
None	200 (36.6)
A little	172 (31.5)
Sometime	122 (22.3)
Most of the time	42 (7.7)
All the time	10 (1.8)
**About how often did you feel that everything was an effort?**	**546**
None	179 (32.8)
A little	162 (29.7)
Sometime	119 (21.8)
Most of the time	73 (13.4)
All the time	13 (2.4)
**About how often did you feel so sad that nothing could cheer you up?**	**546**
None	297 (54.4)
A little	149 (27.3)
Sometime	74 (13.6)
Most of the time	23 (4.2)
All the time	3 (0.5)
**About how often did you feel worthless?**	**547**
None	328 (60.0)
A little	116 (21.2)
Sometime	70 (12.8)
Most of the time	28 (5.1)
All the time	5 (0.9)
**K10 score (total)**	**547**
Mean (±SD)	19.6 (7.6)
Range	10 to 45
**Level of psychological distress (K10 categories)**	**547**
Low (score 10–15)	205 (37.5)
Moderate (score 16–21)	160 (29.3)
High (score 22–29)	111 (20.3)
Very high (score 30–50)	71 (13.0)

**Table 3:** Level of fear of COVID-19 among the study participants

Fear of COVID-19 Scale (FCV-19S) individual items	Total, ***n***(%)
**I am most afraid of COVID-19**	**549**
Strongly disagree	52 (9.5)
Somewhat disagree	72 (13.1)
Neither agree nor disagree	125 (22.8)
Somewhat agree	207 (37.7)
Strongly agree	93 (16.9)
**It makes me uncomfortable to think about COVID-19**	**549**
Strongly disagree	85 (15.5)
Somewhat disagree	89 (16.2)
Neither agree nor disagree	99 (18.0)
Somewhat agree	203 (37.0)
Strongly agree	73 (13.3)
**My hands become clammy when I think about COVID-19**	**549**
Strongly disagree	294 (53.6)
Somewhat disagree	87 (15.8)
Neither agree nor disagree	93 (16.9)
Somewhat agree	59 (10.7)
Strongly agree	16 (2.9)
**I am afraid of losing my life because of COVID-19**	**549**
Strongly disagree	172 (31.3)
Somewhat disagree	76 (13.8)
Neither agree nor disagree	125 (22.8)
Somewhat agree	123 (22.4)
Strongly agree	53 (9.7)
**When watching news and stories about COVID-19 on social media, I become nervous or anxious**	**549**
Strongly disagree	76 (13.8)
Somewhat disagree	70 (12.8)
Neither agree nor disagree	93 (16.9)
Somewhat agree	222 (40.4)
Strongly agree	88 (16.0)
**I cannot sleep because I’m worrying about getting COVID-19**	**549**
Strongly disagree	299 (54.5)
Somewhat disagree	79 (14.4)
Neither agree nor disagree	95 (17.3)
Somewhat agree	69 (12.6)
Strongly agree	7 (1.3)
**My heart races or palpitates when I think about getting COVID-19**	**549**
Strongly disagree	285 (51.9)
Somewhat disagree	86 (15.7)
Neither agree nor disagree	93 (16.9)
Somewhat agree	76 (13.8)
Strongly agree	9 (1.6)
**FCV-19S score (total)**	**549**
Mean (±SD)	18.4 (6.5)
Range	7 to 35
**Level of fear of COVID-19 (FCV-19S categories)**	**549**
Low (score 7–21)	374 (68.1)
High (score 22–35)	175 (31.9)

**Table 4:** Coping during COVID-19 pandemic among the study participants

Brief Resilient Coping Scale (BRCS) individual items	Total, ***n***(%)
**I look for creative ways to alter difficult situations**	**549**
Does not describe me at all	110 (20.0)
Does not describe me	255 (46.4)
Neutral	123 (22.4)
Describes me	35 (6.4)
Describes me very well	26 (4.7)
**Regardless of what happens to me, I believe I can control my reaction to it**	**549**
Does not describe me at all	133 (24.2)
Does not describe me	254 (46.3)
Neutral	109 (19.9)
Describes me	37 (6.7)
Describes me very well	16 (2.9)
**I believe I can grow in positive ways by dealing with difficult situations**	**549**
Does not describe me at all	217 (39.5)
Does not describe me	254 (46.3)
Neutral	62 (11.3)
Describes me	12 (2.2)
Describes me very well	4 (0.7)
**I actively look for ways to replace the losses I encounter in life**	**549**
Does not describe me at all	128 (23.3)
Does not describe me	243 (44.3)
Neutral	139 (25.3)
Describes me	34 (6.2)
Describes me very well	5 (0.9)
**BRCS score (total)**	**549**
Mean (±SD)	8.4 (2.6)
Range	4 to 18
**Level of coping (BRCS categories)**	**549**
Low resilient copers (score 4–13)	534 (97.3)
Medium resilient copers (score 14–16)	12 (2.2)
High resilient copers (score 17–20)	3 (0.5)

### Psychological distress

Univariate analyses showed that younger participants, participants living without family members, females, those with pre-existing mental health conditions, those that did not identify themselves as frontline workers, those with increased smoking and alcohol drinking in the last four weeks, those self-isolating, those who used health service in general or used health service to overcome COVID-19 related stress in the last four weeks and those with a higher level of fear of COVID-19 were more likely to develop moderate to very high levels of psychological distress compared to their counterparts (Table 5). However, when potential confounders were adjusted, being female (AOR 1.74, 95% CIs 1.15–2.65, *p* = 0.009), pre-existing mental health conditions (AOR 3.13, 95% CIs 1.12–8.75, *p* = 0.029), high risk behaviours such as increased smoking (AOR 8.66, 95% CIs 1.08–69.1, *p* = 0.042) and increased alcohol drinking (AOR 2.39, 95% CIs 1.05–5.47, *p* = 0.038) in the past four weeks and higher levels of fear of COVID-19 (AOR 2.93, 95% CIs 1.83–4.67, *p* = 0.000) were associated with moderate to very high levels of psychological distress. Conversely, being older (60+ years) (AOR 0.29, 95% CIs 0.11–0.74, *p* = 0.010) and being a frontline or essential service worker (AOR 0.64, 95% CIs 0.42–0.96, *p* = 0.032) were associated with low levels of psychological distress (Table 5).

**Table 5:** Factors associated with high psychological distress among the study population (based on K10 score)

Characteristics	Moderate to Very High (score 16–50), ***n***(%)	Low (score 10–15), ***n***(%)	Unadjusted analyses			Adjusted analyses		
			***p***	OR	95% CIs	***p***	AOR	95% CIs
Total study participants	341	205						
**Age groups**	**313**	**187**						
18–29 years	72 (23.0)	25 (13.4)		1			1	
30–59 years	224 (71.6)	139 (74.3)	***0.023***	***0.56***	***0.34–0.92***	0.358	0.75	0.41–1.37
60+ years	17 (5.4)	23 (12.3)	***0.001***	***0.26***	***0.12–0.56***	***0.010***	***0.29***	***0.11–0.74***
**Gender**	**341**	**205**						
Male	106 (31.1)	98 (47.8)		1			1	
Female	235 (68.9)	107 (52.2)	***0.000***	***2.03***	***1.42–2.90***	***0.009***	***1.74***	***1.15–2.65***
**Living status**	**339**	**205**						
Live without family members (on your own/shared house/others)	87 (25.7)	36 (17.6)		1			1	
Live with family members (partner and/or children)	252 (74.3)	169 (82.4)	***0.029***	***0.62***	***0.40–0.95***	0.210	0.71	0.41–1.22
**Born in Australia**	**342**	**205**						
No	200 (58.5)	126 (61.5)		1			1	
Yes	142 (41.5)	79 (38.5)	0.491	1.13	0.79–1.61	0.623	1.12	0.70–1.80
**Completed level of education**	**342**	**205**						
Grade 1–12	43 (12.6)	26 (12.7)		1			1	
Trade/Certificate/Diploma	62 (18.1)	22 (10.7)	0.129	1.7	0.86–3.39	0.106	1.89	0.87–4.09
Bachelor and above	237 (69.3)	157 (76.6)	0.734	0.91	0.54–1.55	0.974	1.01	0.52–1.95
**Current employment condition**	**326**	**195**						
Unemployed/Home duties	44 (13.5)	19 (9.7)		1			1	
Jobs affected by COVID-19 (lost job/working hours reduced/afraid of job loss)	59 (18.1)	22 (11.3)	0.693	1.16	0.56–2.40	0.948	1.02	0.46–2.32
Have an income source (employed/Government benefits)	223 (68.4)	154 (79.0)	0.110	0.63	0.35–1.11	0.113	0.59	0.30–1.14
**Perceived distress due to change of employment status**	**118**	**33**						
A little to none	27 (22.9)	10 (30.3)		1			1	
Moderate to a great deal	91 (77.1)	23 (69.7)	0.383	1.47	0.62–3.45	0.399	1.58	0.55–4.54
**Self-identification as a frontline or essential service worker**	**342**	**205**						
No	213 (62.3)	101 (49.3)		1			1	
Yes	129 (37.7)	104 (50.7)	***0.003***	***0.59***	***0.41–0.83***	***0.032***	***0.64***	***0.42–0.96***
**COVID-19 impacted financial situation**	**341**	**205**						
No	137 (40.2)	91 (44.4)		1			1	
Yes	204 (59.8)	114 (55.6)	0.334	1.19	0.84–1.69	0.534	1.14	0.76–1.72
**Co-morbidities**	**342**	**205**						
No	169 (49.4)	104 (50.7)		1			1	
Psychiatric/Mental health issues	36 (10.5)	5 (2.4)	***0.003***	***4.43***	***1.69–11.7***	***0.029***	***3.13***	***1.12–8.75***
Other co-morbidities*	137 (40.1)	96 (46.8)	0.476	0.88	0.61–1.26	0.613	1.11	0.73–1.69
**Smoking**	**267**	**158**						
Never smoker	226 (84.6)	144 (91.1)		1			1	
Ever smoker (Daily/Non-daily/Ex)	41 (15.4)	14 (8/9)	0.054	1.87	0.98–3.55	0.054	2.03	0.99–4.17
**Increased smoking over the last 4 weeks**	**41**	**14**						
No	22 (53.7)	12 (85.7)		1			1	
Yes	19 (46.3)	2 (14.3)	***0.046***	***5.18***	***1.03–26.1***	***0.042***	***8.66***	***1.08–69.1***
**Current alcohol drinking (last 4 weeks)**	**341**	**205**						
No	206 (60.4)	138 (67.3)		1			1	
Yes	135 (39.6)	67 (32.7)	0.106	1.35	0.94–1.94	0.196	1.35	0.85–2.15
**Increased alcohol drinking over the last 4 weeks**	**134**	**65**						
No	84 (62.7)	54 (83.1)		1			1	
Yes	50 (37.3)	11 (16.9)	***0.004***	***2.92***	***1.40–6.11***	***0.038***	***2.39***	***1.05–5.47***
**Provided care to a family member/patient with known/suspected case of COVID-19**	**321**	**195**						
No	281 (82.2)	170 (82.9)		1			1	
Yes	61 (17.8)	35 (17.1)	0.820	1.05	0.67–1.67	0.841	1.05	0.63–1.76
**Experience related to COVID-19 pandemic**	**342**	**205**						
I have been self-isolating prior to receiving negative result for COVID-19	52 (15.2)	19 (9.3)	***0.048***	***1.76***	***1.01–3.06***	0.082	1.75	0.93–3.30
**Healthcare service use in the last 4 weeks**	**342**	**205**						
No	255 (74.6)	178 (86.8)		1			1	
Visited healthcare providers in person	47 (13.7)	17 (8.3)	***0.028***	***1.93***	***1.07–3.47***	0.983	1.01	0.30–3.41
Telehealth consultation with healthcare providers/National helpline	40 (11.7)	10 (4.9)	***0.005***	***2.79***	***1.36–5.73***	0.408	0.46	0.07–2.93
**Level of fear of COVID-19 (FCV-19S categories)**	**342**	**205**						
Low (score 7–21)	207 (60.5)	166 (81.0)		1			1	
High (score 22–35)	135 (39.5)	39 (19.0)	***0.000***	***2.78***	***1.84–4.19***	***0.000***	***2.93***	***1.83–4.67***
**Level of coping (BRCS categories)**	**342**	**205**						
Low resilient copers (score 4–13)	333 (97.4)	200 (97.6)		1			1	
Medium to high resilient copers (score 14–20)	9 (2.6)	5 (2.4)	0.89	1.08	0.36–3.27	0.741	1.22	0.38–3.87
**Healthcare service use to overcome COVID-19 related stress in the last 4 weeks**	**342**	**205**						
No	306 (89.5)	203 (99.0)		1			1	
Yes	36 (10.5)	2 (6.9)	***0.001***	***11.9***	***2.84–50.1***	0.004	20.2	2.64–154

*Adjusted for: age, gender, living status, born in Australia, education and employment*

^*^ Cardiac diseases/Stroke/Hypertension/Hyperlipidaemia/Diabetes/Cancer/Chronic respiratory illness

### Level of fear

Univariate analyses showed that being middle aged, living with family members, being born overseas, unemployed, those reporting moderate to a great deal of distress due to a change of employment status, those who did not identify themselves as frontline workers, those whose financial situation was impacted, those with current and increased alcohol drinking in the last four weeks, those who used health service to overcome COVID19 related stress in the last four weeks and those with a moderate to very high level of psychological distress were more likely to have higher levels of fear of COVID-19 than other study participants. After adjusting for potential confounders, being female (AOR 1.56, 95% CIs 1.00–2.45, *p* = 0.052), aged 30–59 years old (AOR 2.29, 95% CIs 1.21–4.35, *p* = 0.011), perceived moderate to a great deal of distress due to a change of employment status (AOR 4.14, 95% CIs 1.39–12.4, *p* = 0.011), providing care to known or suspected cases of COVID-19 (AOR 3.64, 95% CIs 1.54–8.58, *p* = 0.003), increased alcohol drinking over the last four weeks (AOR 3.64, 95% CIs 1.54–8.58, *p* = 0.003), having medium to very high levels of distress based on the K10 scale (AOR 2.90, 95% CIs 1.82–5.62, *p* = 0.000), and health service use to overcome COVID-19 related stress in the last four weeks (AOR 3.54, 95% CIs 1.57–7.99, *p* = 0.002) were associated with higher levels of fear. On the other hand, being born in Australia (AOR 0.35, 95% CIs 0.21–0.58, *p* = 0.000), having a source of income (AOR 0.51, 95% CIs 0.27–0.95, *p* = 0.033), and alcohol drinking in the last four weeks (AOR 0.45, 95% CIs 0.27–0.74, *p* = 0.002) were associated with lower level of fear (Table 6).

**Table 6:** Factors associated with levels of fear of COVID-19 among the study population (based on FCV-19S score)

Characteristics	High (score 22–35), ***n***(%)	Low (score 7–21), ***n***(%)	Unadjusted analyses			Adjusted analyses		
			***p***	OR	95% CIs	***p***	AOR	95% CIs
Total study participants	175	374						
**Age groups**	**162**	**339**						
18–29 years	22 (13.6)	75 (22.1)		1			1	
30–59 years	131 (80.9)	233 (68.7)	***0.014***	***1.92***	***1.14–3.23***	***0.011***	***2.29***	***1.21–4.35***
60+ years	9 (5.6)	31 (9.1)	0.982	0.99	0.41–2.39	0.551	1.37	0.49–3.84
**Gender**	**174**	**374**						
Male	61 (35.1)	145 (38.8)		1			1	
Female	113 (64.9)	229 (61.2)	0.404	1.17	0.81–1.71	0.052	***1.56***	***1.00–2.45***
**Living status**	**174**	**371**						
Live without family members (on your own/shared house/others)	29 (16.7)	95 (25.6)		1			1	
Live with family members (partner and/or children)	145 (83.3)	276 (74.4)	***0.020***	***1.72***	***1.08–2.73***	0.502	1.22	0.69–2.16
**Born in Australia**	**175**	**374**						
No	127 (72.6)	200 (53.5)		1			1	
Yes	48 (27.4)	174 (46.5)	***0.000***	***0.43***	***0.29–0.64***	***0.000***	***0.35***	***0.21–0.58***
**Completed level of education**	**175**	**373**						
Grade 1–12	19 (10.9)	50 (13.4)		1			1	
Trade/Certificate/Diploma	26 (14.9)	59 (15.8)	0.679	1.16	0.58–2.34	0.414	0.72	0.32–1.59
Bachelor and above	130 (74.3)	264 (70.8)	0.372	1.30	0.74–2.29	0.129	0.58	0.29–1.17
**Current employment condition**	**169**	**354**						
Unemployed/Home duties	28 (16.6)	35 (9.9)		1			1	
Jobs affected by COVID-19 (lost job/working hours reduced/afraid of job loss)	40 (23.7)	42 (11.9)	0.604	1.19	0.62–2.30	0.517	1.28	0.6–2.69
Have an income source (employed/Government benefits)	101 (59.8)	277 (78.2)	***0.005***	***0.46***	***0.26–0.79***	***0.033***	***0.51***	***0.27–0.95***
**Perceived distress due to change of employment status**	**60**	**92**						
A little to none	6 (10.0)	31 (33.7)		1			1	
Moderate to a great deal	54 (90.0)	61 (66.3)	***0.002***	***4.57***	***1.77–11.8***	***0.011***	***4.14***	***1.39–12.4***
**Self-identification as a frontline or essential service worker**	**175**	**374**						
No	113 (64.6)	202 (54.0)		1			1	
Yes	62 (35.4)	172 (46.0)	***0.020***	***0.64***	***0.44–0.93***	0.065	0.66	0.43–1.03
**COVID-19 impacted financial situation**	**175**	**372**						
No	60 (34.3)	169 (45.4)		1			1	
Yes	115 (65.7)	203 (54.6)	***0.014***	***1.60***	***1.10–2.32***	0.297	1.26	0.82–1.95
**Co-morbidities**	**175**	**374**						
No	91 (52.0)	184 (49.2)		1			1	
Psychiatric/Mental health issues	12 (6.9)	29 (7.8)	0.626	0.84	0.41–1.72	0.952	1.02	0.49–2.12
Other co-morbidities*	72 (41.1)	161 (43.0)	0.598	0.90	0.62–1.32	0.698	1.09	0.69–1.72
**Smoking**	**138**	**288**						
Never smoker	117 (84.8)	254 (88.2)		1			1	
Ever smoker (Daily/Non-daily/Ex)	21 (15.2)	34 (11.8)	0.326	1.34	0.75–2.41	0.430	1.31	0.67–2.59
**Increased smoking over the last 4 weeks**	**21**	**34**						
No	12 (57.1)	22 (64.7)		1			1	
Yes	9 (42.9)	12 (35.3)	0.575	1.38	0.45–4.19	0.902	1.09	0.29–4.14
**Current alcohol drinking (last 4 weeks)**	**174**	**374**						
No	131 (75.3)	215 (57.5)		1			1	
Yes	43 (24.7)	159 (42.5)	***0.000***	***0.44***	***0.30–0.66***	***0.002***	***0.45***	***0.27–0.74***
**Increased alcohol drinking over the last 4 weeks**	**41**	**158**						
No	20 (48.8)	118 (74.7)		1			1	
Yes	21 (51.2)	40 (25.3)	***0.002***	***3.10***	***1.52–6.30***	***0.003***	***3.64***	***1.54–8.58***
**Provided care to a family member/patient with known/suspected case of COVID-19**	**175**	**374**						
No	144 (82.3)	308 (82.4)		1			1	
Yes	31 (17.7)	66 (17.6)	0.985	1.01	0.63–1.61	0.929	0.98	0.57–1.67
**Healthcare service use in the last 4 weeks**	**175**	**374**						
No	133 (76.0)	302 (80.7)		1			1	
Visited healthcare providers in person	21 (12.0)	43 (11.5)	0.718	1.11	0.63–1.94	0.410	1.52	0.56–4.15
Telehealth consultation with healthcare providers/National helpline	21 (12.0)	29 (7.8)	0.103	1.64	0.90–2.99	0.163	3.21	0.62–16.5
**Level of psychological distress (K10 categories)**	**174**	**373**						
Low (score 10–15)	39 (22.4)	166 (44.5)		1			1	
Medium to Very high (score 16–50)	135 (77.6)	207 (55.5)	***0.000***	***2.78***	***1.84–4.19***	***0.000***	***2.90***	***1.82–4.62***
**Level of coping (BRCS categories)**	**175**	**374**						
Low resilient copers (score 4–13)	170 (97.1)	364 (97.3)		1			1	
Medium to high resilient copers (score 14–20)	5 (2.9)	10 (2.7)	0.902	1.07	0.36–3.18	0.953	0.96	0.28–3.32
**Healthcare service use to overcome COVID-19 related stress in the last 4 weeks**	**175**	**374**						
No	157 (89.7)	354 (94.7)		1			1	
Yes	18 (10.3)	20 (5.3)	***0.037***	***2.03***	***1.04–3.94***	***0.002***	***3.54***	***1.57–7.99***

*Adjusted for: age, gender, living status, born in Australia, education and employment*

^*^ Cardiac diseases/Stroke/Hypertension/Hyperlipidaemia/Diabetes/Cancer/Chronic respiratory illness

### Coping strategies

When medium to high resilient copers were compared with low resilient copers based on the BRCS scale, the only significant factor was visiting healthcare providers in person in the last four weeks (OR 4.01, 95% CIs 1.30–12.4, *p* = 0.016). However, it did not remain statistically significant when sociodemographic variables were adjusted as potential confounders. The study respondents mentioned a varied range of experiences/activities to cope up with the stress from COVID-19. The most emerging themes included engaging in daily exercise, yoga and meditation; watching movies; listening to music and reading books; spending time with kids and partners or focusing on family members; gardening; making phone calls to friends and loved ones; engaging in hobbies one was used to; cleaning home and hand washing; ensuring safe distancing; more prayers and connecting to God; working from home; cooking and eating more; drinking and smoking; not thinking too much about the situation; not watching news/statistics about COVID-19; visiting GPs or psychologist to allay fears and distress and finally, doing nothing special.

## Discussion

This cross-sectional study was one of the earliest carried out among residents of Australia with a view to assess the extent of and identify factors associated with psychological distress, level of fear and coping strategies during the COVID-19 pandemic. Being female and increased alcohol drinking in the past four weeks were associated with higher psychological distress and higher levels of fear of COVID-19 in this study. In addition, higher psychological distress was associated with pre-existing mental health conditions, increased smoking in the last four weeks and higher levels of fear of COVID-19 while lower psychological distress was associated with being older (60+ years) and being a frontline or essential service worker. A higher level of fear was associated with being 30–59 years old, perceived distress due to change of employment status, providing care to known or suspected case of COVID-19 and having higher level of psychological distress. On the other hand, a lower level of fear was associated with being born in Australia, having a source of income and alcohol drinking in the last four weeks. Visiting healthcare providers in person in the last four weeks was also found to be associated with coping during the COVID-19 pandemic.

Previous research has revealed a profound and wide range of psychosocial impacts on people at the individual and community level during outbreaks of infection [25]. However, it will be somewhat early to predict similar results emanating during the peak of the COVID-19 epidemic, with the uncertainty surrounding an outbreak of such unparalleled magnitude. A recent study carried out in 194 cities in China showed 53.8% respondents rating psychological impact as moderate to severe [14]. This finding coincides with our study as it raised almost similar levels (moderate to very high level) of psychological distress (62.6%) among the study participants. A study from the UK showed that the prevalence of poor mental health was 37% amongst participants who were in isolation or maintaining social distancing, where poor mental health was defined as having moderate to severe depressive symptoms and anxiety [26]. Similarly, the Australian Bureau of Statistics had been collecting COVID-19 impact survey data fortnightly involving a representative sample of over 1000 adults across Australia, and findings indicated twice as many adults experiencing anxiety, nervousness and restlessness compared to pre-COVID surveillance data [27].

Evidence suggests that pre-existing anxiety disorders, existing health anxiety (those who worry excessively about having or contracting illnesses), and other mental health disorders (e.g., depression and post-traumatic stress) are at risk of experiencing higher anxiety levels during the COVID-19 outbreak [28]. Similarly, our study showed a significantly higher level of psychological distress among participants having pre-existing co-morbidities such as psychiatric or mental health issues. Individuals who were self-isolating prior to receiving negative results for COVID-19 also showed heightened psychological stress and this was corroborated by another study, which identified anxiousness and feelings of guilt by the quarantined persons [14]. In addition, a sense of stigma from other family members or friends might have contributed to such high levels of distress [29]. Findings related to self-identification as a frontline or essential service worker exhibiting a lower level of stress in the present study was somewhat incongruous with other initial studies, which showed a significant mental health burden on frontline healthcare workers during pandemics [30]. A recent systematic review of 59 studies with 54,707 participants showed that one or two of every five healthcare professionals reported anxiety, depression, distress and/or sleep problems during the current COVID-19 pandemic, which were primarily associated with increased workload [31]. Due to the small number of participants, our study could not conduct subgroup analyses focusing on frontline healthcare providers and our findings of lower levels of distress among these groups could be due to the prolonged exposure of the pandemic period, being accustomed with service provision as frontline workers and/or availability of personal protective equipment.

The COVID-19 pandemic also led to maladaptive behaviours, including increased smoking and alcohol intake due to stress and social isolation [4]. Previous studies also found that patients with a history of smoking are at higher risk of severe COVID disease, and those admitted to intensive care, may require ventilation [32]. Our study has found significant association between increased smoking as well as alcohol drinking and higher psychological distress. Evidence from previous studies indicated community-wide disasters being associated with a number of behavioural health outcomes including increased mental health concerns and escalations in the use of alcohol [33, 34].

Social distancing, stay at home orders and quarantine measures may lead to boredom, uncertainty and disruption to routines and distress resulting in elevated psychological distress as found in our present study. Increased alcohol consumption might also be explained as a coping mechanism for the perceived distress as many self-medication hypotheses posit use of substances like alcohol for relieving distress [35]. Our study results are also consistent with the findings from a literature review which documented an increase in alcohol consumption for some populations, particularly men, because of added mental stress due to uncertainty about the future due to COVID-19, and economic and employment concerns experienced as a result of the pandemic [36]. The same can be stated regarding increased smoking, as most nicotine consumers reported using nicotine products as their main stress and anxiety coping mechanism. A multi-country study conducted in Italy, India, South Africa, the United Kingdom and the United States have revealed that during the COVID-19 pandemic, cigarette smokers have been buying more cigarettes than usual triggered by the fear that stores might run out of stock or be closed during lock down [37]. Another study from the USA reported that one-third of the participants increased their tobacco use during the COVID-19 pandemic and subsequent ‘stay-at-home’ orders; that negative behaviour was associated with sociodemographic variables and depression [38].

Our study showed females had higher distress and fear of COVID-19, which was consistent with studies from China [39], Italy [40] and the USA [41] suggesting that female gender was a consistent predictor for psychological distress. A host of reasons can be postulated for this as females disproportionately share the larger percentage of caregiving roles, in both formal and informal sectors. They also serve as the primary caregivers more frequently, within a household, which may further accentuate their anxiety and stress in a pandemic situation [42]. The abovementioned Chinese studies also showed that young adults (aged 18–30 years) exhibited the highest level of psychological distress, which was consistent with our study findings. Such distress could be correlated with an increased use of social media, as young participants may watch and listen to much more negative news, which would then intensify their feelings of anxiety and depression in times of crisis.

Higher levels of fear amongst middle-aged participants in our study were more likely due to being part of the workforce with possible financial uncertainly in the event of future job loss. This could also be a possible explanation of accentuation of psychological distress, and therefore, participants utilised more healthcare services (by physical visits or through telehealth) to overcome the COVID-19 related stress during the study period. Like previous studies, our study also found that fear of COVID-19 was a prominent risk factor for the onset and maintenance of increased alcohol consumption [36]. This explains why participants used alcohol as part of their neuro-adaptations and coping response to the stress (induced by fear) because of the pandemic and social isolation [43].

The results of our study further illuminated additional aspects of the factors relating to fear of COVID-19. Level of education did not have any impact on the fear of COVID-19. Similarly, participants had no association between fear and having any existing comorbidities or increased healthcare utilisation. No association was also found between fear and providing care to family or patients with known or suspected COVID-19. There could be multiple possible explanations for this finding. First, a third of the participants, being frontline essential workers, somehow accepted the situation and their role as caregivers or service providers to the family and population they served, respectively. Second, the pandemic was not regarded as severe in intensity in Australia during the study period (June 2020). Third, it could have been that participants were less aware of the severity of the virus; and finally, trust in the initiatives taken by the Australian Government, including stage 3 restrictions with banning of non-essential travel between and within the states and social isolation to prevent the exponential spread of the virus. Participants born in Australia also experienced less fear compared to those not born in Australia, which might be related to better knowledge about the health system, support networks and stronger coping by the Australia born residents. Another study from Australia reported that self-perceived probability of losing jobs was greater amongst people who were born overseas in a non-English speaking country compared to those who were born in Australia, which could also explain the higher psychological distress in non-Australian born residents in our study [44]. However, it was beyond the scope of this study to examine all the relevant factors. Our study demonstrated that frontline or essential workers were less fearful than their counterparts, which could be explained by the availability of increased testing and personal protective equipment for the health care and frontline workers.

More respondents in our study completed bachelor and above qualifications, possibly due to higher number of front line or essential service workers taking part in this survey, who would presumably had higher education than others. According to the International Labour Market (ILO), almost 25 million jobs could be lost worldwide due to COVID-19 [45]. However, pervasive job loss was not evident among our study population as one-third of them were working as frontline or essential service workers who would not necessarily lose jobs during such a crisis, and other participants might have benefitted from the employment support initiative by the Australian Government like *jobseeker or jobkeeper payment* [46, 47]. Accordingly, employment status of our study participants might not reflect the true job loss situation in Australia due to the ongoing pandemic of COVID-19.

### Limitations

The survey responses in this study were predominantly from Victoria although the survey link was shared across all the states in Australia through various social media platforms and emails. This could be explained by the researchers’ use of snowball sampling technique as community acquaintances and their accessibility to GP clinics/ allied health service facilities in Victoria than in the other Australian states. Therefore, findings of this study might be more generalisable to the State of Victoria rather than across Australia, and it was also likely that distressed individuals had responded to our survey resulting in selection bias. A significant number of participants in the study were not born in Australia, which is a reflection of the country’s multiculturalism. According to the Australian Bureau of Statistics, almost 30% of Australians were born overseas, increasing the cultural diversity of Australia’s population [48]. More females than males participated in the survey, which could be due to more frequent visits to healthcare providers by females than males [49], and increased number of female workforce being employed in the frontline healthcare or essential service facilities/outlets [50]. Findings of our study were limited to people who could access online platforms to participate; hence, generalizability was limited to internet-literate people. Considering the restrictions of movement and social distancing, an online survey was the only viable option during the pandemic to address our research objectives. However, the strength of the study was the achievement of the target sample size within the crisis period; hence the study had significant power to test the hypotheses.

Social distancing and self-isolation due to the current COVID-19 pandemic were likely to be stressful for people. Therefore, it was also important to understand responses from our study participants regarding their coping strategies, considering some groups could be more vulnerable than others to the psychosocial effects of the pandemic. Findings of our study were supported by prior research outlining coping activities like social connection with families and friends, limiting exposure to pandemic-related news, maintaining adequate sleep, nutrition, exercise, and practicing meditation (mindfulness) [51].

## Conclusions

This study identified individuals who were at higher risk of distress and fear during the COVID-19 pandemic situation, specifically in the State of Victoria, Australia. People with higher psychological distress increased their use of smoking and alcohol during the pandemic period, which warrants targeting behavioural interventions specifically for those groups and incorporating information on available support services to quit smoking and reduce alcohol use in health awareness campaigns during such pandemic periods of uncertainty.

People with pre-existing mental health conditions were also more likely to experience higher psychological distress, which could worsen their overall wellbeing. An automated alert from primary healthcare providers to those vulnerable individuals for a follow-up visit would be invaluable in managing their distress. In addition, our study indicated that females were more vulnerable to psychological distress, and as such socio-cultural contexts rather than biomedical contexts influenced their mental health, which should be recognised and supported accordingly. Specific interventions to support the mental wellbeing of higher risk individuals as identified in this study should be considered in addition to existing resources within primary healthcare settings. Innovative technologies such as interactive mobile apps to support mental wellbeing can be developed and tested for effectiveness in future experimental studies.

## Data Availability

The datasets used and/or analysed during the current study are available from the corresponding author on reasonable request.

## References

[1] Worldometer. COVID-19 Coronavirus Pandemic [Available from: https://www.worldometers.info/coronavirus.

[2] Australian Government Department of Health. Coronavirus (COVID-19) current situation and case numbers [Available from: https://www.health.gov.au/news/health-alerts/novel-coronavirus-2019-ncov-health-alert/coronavirus-covid-19-current-situation-and-case-numbers.

[3] Van Rheenen TE, Meyer D, Neill E, Phillipou A, Tan EJ, Toh WL (2020). Mental health status of individuals with a mood-disorder during the COVID-19 pandemic in Australia: initial results from the COLLATE project. J Affect Disord.

[4] Stanton R, To QG, Khalesi S, Williams SL, Alley SJ, Thwaite TL, et al. Depression, Anxiety and Stress during COVID-19: Associations with Changes in Physical Activity, Sleep, Tobacco and Alcohol Use in Australian Adults. Int J Environ Res Public Health. 2020;17(11). 10.3390/ijerph17114065 PMC7312903 32517294

[5] Roy Morgan. Roy Morgan Unemployment Profile Melbourne, Australia. 2020 [Available from: http://www.roymorgan.com/morganpoll/unemployment/estimates-detailed.

[6] Ministers Department of Health: Australian Government. COVID-19: Whole of population telehealth for patients, general practice, primary care and other medical services 2020 [Available from: https://www.health.gov.au/ministers/the-hon-greg-hunt-mp/media/covid-19-whole-of-population-telehealth-for-patients-general-practice-primary-care-and-other-medical-services.

[7] Thombs BD, Bonardi O, Rice DB, Boruff JT, Azar M, He C, et al. Curating evidence on mental health during COVID-19: a living systematic review. J Psychosom Res. 2020;110113. 10.1016/j.jpsychores.2020.110113 PMC7185913 32354463

[8] Furlong Y, Finnie T. Culture counts: the diverse effects of culture and society on mental health amidst COVID-19 outbreak in Australia. Ir J Psychol Med. 2020:1–13. 10.1017/ipm.2020.37 PMC7373827 32406358

[9] Gunnell D, Appleby L, Arensman E, Hawton K, John A, Kapur N (2020). Suicide risk and prevention during the COVID-19 pandemic. Lancet Psychiatry.

[10] Australian Bureau of Statistics. Population clock 2020 [Available from: https://www.abs.gov.au/ausstats/abs%40.nsf/94713ad445ff1425ca25682000192af2/1647509ef7e25faaca2568a900154b63?OpenDocument.

[11] Australian Bureau of Statistics. 4326.0 - National Survey of Mental Health and Wellbeing: Summary of Results, 2007 2008 [Available from: https://www.abs.gov.au/AUSSTATS/abs@.nsf/Lookup/4326.0Main+Features32007?OpenDocument.

[12] Huang Y, Zhao N. Generalized anxiety disorder, depressive symptoms and sleep quality during COVID-19 epidemic in China: a web-based cross-sectional survey. medRxiv. 2020;In Press. 10.1016/j.psychres.2020.112954 PMC7152913 32325383

[13] Cai H, Tu B, Ma J, Chen L, Fu L, Jiang Y, et al. Psychological Impact and Coping Strategies of Frontline Medical Staff in Hunan Between January and March 2020 During the outbreak of coronavirus disease 2019 (COVID-19) in Hubei, China. Med Sci Monit. 2020:26. 10.12659/MSM.924171 PMC7177038 32291383

[14] Wang C, Pan R, Wan X, Tan Y, Xu L, Ho CS, et al. Immediate Psychological Responses and Associated Factors during the Initial Stage of the 2019 Coronavirus disease (COVID-19) epidemic among the general population in China. Int J Environ Res Public Health. 2020:17(5). 10.3390/ijerph17051729 PMC7084952 32155789

[15] Kang L, Ma S, Chen M, Yang J, Wang Y, Li R, et al. Impact on mental health and perceptions of psychological care among medical and nursing staff in Wuhan during the 2019 novel coronavirus disease outbreak: A cross-sectional study. Brain, Behavior, and Immunity. 2020;In Press. 10.1016/j.bbi.2020.03.028 PMC7118532 32240764

[16] Furukawa TA, Kessler RC, Slade T, Andrews G (2003). The performance of the K6 and K10 screening scales for psychological distress in the Australian National Survey of mental health and well-being. Psychol Med.

[17] Ahorsu DK, Lin CY, Imani V, Saffari M, Griffiths MD, Pakpour AH. The fear of COVID-19 scale: development and initial validation. Int J Ment Health Addict. 2020:1–9. 10.1007/s11469-020-00270-8 PMC7100496 32226353

[18] Sinclair VG, Wallston KA (2004). The development and psychometric evaluation of the brief resilient coping scale. Assessment..

[19] Kessler RC, Andrews G, Colpe LJ, Hiripi E, Mroczek DK, Normand SL (2002). Short screening scales to monitor population prevalences and trends in non-specific psychological distress. Psychol Med.

[20] Enticott JC, Lin E, Shawyer F, Russell G, Inder B, Patten S (2018). Prevalence of psychological distress: how do Australia and Canada compare?. Aust N Z J Psychiatry.

[21] Bitan DT, Grossman-Giron A, Bloch Y, Mayer Y, Shiffman N, Shlomo M (2020). Fear of COVID-19 scale: Psychometric characteristics, reliability and validity in the Israeli population. Psychiatry Res.

[22] Winter T, Riordan BC, Pakpour AH, Griffiths MD, Mason A, Poulgrain JW, et al. Evaluation of the English version of the fear of COVID-19 scale and its relationship with behavior change and political beliefs. Int J Ment Health Addict. 2020:1–11. 10.1007/s11469-020-00342-9 PMC7295324 32837431

[23] Kocalevent RD, Zenger M, Hinz A, Klapp B, Brahler E (2017). Resilient coping in the general population: standardization of the brief resilient coping scale (BRCS). Health Qual Life Outcomes.

[24] López-Pina J-A, Meseguer-Henarejos A-B, Gascón-Cánovas J-J, Navarro-Villalba D-J, Sinclair VG, Wallston KA (2016). Measurement properties of the brief resilient coping scale in patients with systemic lupus erythematosus using rasch analysis. Health Qual Life Outcomes.

[25] Hall RC, Hall RC, Chapman MJ (2008). The 1995 Kikwit Ebola outbreak: lessons hospitals and physicians can apply to future viral epidemics. Gen Hosp Psychiatry.

[26] Smith L, Jacob L, Yakkundi A, McDermott D, Armstrong NC, Barnett Y (2020). Correlates of symptoms of anxiety and depression and mental wellbeing associated with COVID-19: a cross-sectional study of UK-based respondents. Psychiatry Res.

[27] Australian Bureau of Statistics. 4940.0 - Household Impacts of COVID-19 Survey, 24-29 2020 2020 [Available from: https://www.abs.gov.au/ausstats/abs@.nsf/mf/4940.0.

[28] Black Dog Institute. Mental Health Ramifications of COVID-19: The Australian context. 2020.

[29] Coronavirus Disease 2019 (COVID-19): Epidemiology, Pathogenesis, Diagnosis, and Therapeutics. Saxena SK, editor. Lucknow, India: Springer; 2020.

[30] Pappa S, Ntella V, Giannakas T, Giannakoulis VG, Papoutsi E, Katsaounou P. Prevalence of depression, anxiety, and insomnia among healthcare workers during the COVID-19 pandemic: a systematic review and meta-analysis. Brain Behav Immun. 2020. 10.1016/j.bbi.2020.05.026 PMC7206431 32437915

[31] Muller AE, Hafstad EV, Himmels JPW, Smedslund G, Flottorp S, Stensland SO (2020). The mental health impact of the covid-19 pandemic on healthcare workers, and interventions to help them: a rapid systematic review. Psychiatry Res.

[32] Guan WJ, Ni ZY, Hu Y, Liang WH, Ou CQ, He JX (2020). Clinical characteristics of coronavirus disease 2019 in China. N Engl J Med.

[33] Walsh K, Elliott JC, Shmulewitz D, Aharonovich E, Strous R, Frisch A (2014). Trauma exposure, posttraumatic stress disorder and risk for alcohol, nicotine, and marijuana dependence in Israel. Compr Psychiatry.

[34] Rodriguez LM, Litt DM, Stewart SH (2020). Drinking to cope with the pandemic: the unique associations of COVID-19-related perceived threat and psychological distress to drinking behaviors in American men and women. Addict Behav.

[35] Khantzian EJ (1997). The self-medication hypothesis of substance use disorders: a reconsideration and recent applications. Harv Rev Psychiatry.

[36] Rehm J, Kilian C, Ferreira-Borges C, Jernigan D, Monteiro M, Parry CDH (2020). Alcohol use in times of the COVID 19: implications for monitoring and policy. Drug Alcohol Rev.

[37] Yach D. Tobacco use patterns in five countries during the COVID-19 lockdown. Nicotine Tob Res. 2020. 10.1093/ntr/ntaa097 PMC7313787 32459837

[38] Knell G, Robertson MC, Dooley EE, Burford K, Mendez KS. Health Behavior Changes During COVID-19 Pandemic and Subsequent “Stay-at-Home” Orders. Int J Environ Res Public Health. 2020; 17(17). 10.3390/ijerph17176268 PMC7504386 32872179

[39] Qiu J, Shen B, Zhao M, Wang Z, Xie B, Xu Y (2020). A nationwide survey of psychological distress among Chinese people in the COVID-19 epidemic: implications and policy recommendations. Gen Psychiatr.

[40] Gausman J, Langer A (2020). Sex and gender disparities in the COVID-19 pandemic. J Women's Health (Larchmt).

[41] French MT, Mortensen K, Timming AR (2020). Psychological distress and coronavirus fears during the initial phase of the COVID-19 pandemic in the United States. J Ment Health Policy Econ.

[42] Langer A, Meleis A, Knaul FM, Atun R, Aran M, Arreola-Ornelas H (2015). Women and health: the key for sustainable development. Lancet..

[43] Clay JM, Parker MO (2020). Alcohol use and misuse during the COVID-19 pandemic: a potential public health crisis?. Lancet Public Health.

[44] Biddle N, Edwards B, Gray M, Sollis K. Hardship, distress, and resilience: The initial impacts of COVID-19 in Australia. The ANU Centre for Social Research and Methods; 2020 Apr-2020.

[45] International Labor Organization (ILO). Almost 25 million jobs could be lost worldwide as a result of COVID-19, says ILO 2020 [Available from: https://www.ilo.org/global/about-the-ilo/newsroom/news/WCMS_738742/lang%2D%2Den/index.htm.

[46] Services Australia: Australian Government. JobSeeker Payment 2020 [Available from: https://www.servicesaustralia.gov.au/individuals/services/centrelink/jobseeker-payment.

[47] Government ATOA. JobKeeper Payment 2020 [Available from: https://www.ato.gov.au/General/JobKeeper-Payment/.

[48] Australian Bureau of Statistics. 3105.0.65.001 - Australian Historical Population Statistics, 2016 2019 [Available from: https://www.abs.gov.au/AUSSTATS/abs@.nsf/DetailsPage/3105.0.65.0012016?OpenDocument.

[49] Young AF, Dobson AJ, Byles JE (2001). Determinants of general practitioner use among women in Australia. Soc Sci Med.

[50] Australian Institute of Health and Welfare (AIHW) (2018). 2.3 Who is in the health workforce?.

[51] Ornell F, Schuch JB, Sordi AO, Kessler FHP (2020). “pandemic fear” and COVID-19: mental health burden and strategies. Braz J Psychiatry.

